# Habitat Fragmentation, Variable Edge Effects, and the Landscape-Divergence Hypothesis

**DOI:** 10.1371/journal.pone.0001017

**Published:** 2007-10-10

**Authors:** William F. Laurance, Henrique E. M. Nascimento, Susan G. Laurance, Ana Andrade, Robert M. Ewers, Kyle E. Harms, Regina C. C. Luizão, José E. Ribeiro

**Affiliations:** 1 Smithsonian Tropical Research Institute, Balboa, Republic of Panama; 2 Biological Dynamics of Forest Fragments Project, National Institute for Amazonian Research (INPA), Smithsonian Tropical Research Institute, Manaus, Brazil; 3 Institute of Zoology, Zoological Society of London, London, United Kingdom; 4 Department of Biological Sciences, Louisiana State University, Baton Rouge, Louisiana, United States of America; 5 Department of Botany, National Institute for Amazonian Research (INPA), Manaus, Brazil; University of Kent, United Kingdom

## Abstract

Edge effects are major drivers of change in many fragmented landscapes, but are often highly variable in space and time. Here we assess variability in edge effects altering Amazon forest dynamics, plant community composition, invading species, and carbon storage, in the world's largest and longest-running experimental study of habitat fragmentation. Despite detailed knowledge of local landscape conditions, spatial variability in edge effects was only partially foreseeable: relatively predictable effects were caused by the differing proximity of plots to forest edge and varying matrix vegetation, but windstorms generated much random variability. Temporal variability in edge phenomena was also only partially predictable: forest dynamics varied somewhat with fragment age, but also fluctuated markedly over time, evidently because of sporadic droughts and windstorms. Given the acute sensitivity of habitat fragments to local landscape and weather dynamics, we predict that fragments within the same landscape will tend to converge in species composition, whereas those in different landscapes will diverge in composition. This ‘landscape-divergence hypothesis’, if generally valid, will have key implications for biodiversity-conservation strategies and for understanding the dynamics of fragmented ecosystems.

## Introduction

Habitat fragmentation is among the most important of all threats to global biodiversity [1], [2], and edge effects—diverse physical and biotic alterations associated with the artificial boundaries of fragments—are dominant drivers of change in many fragmented landscapes [2]–[10]. Edge effects can have serious impacts on species diversity and composition, community dynamics, and ecosystem functioning [11]–[17].

Many edge effects are variable in space and time [7], [9], [18]–[21]. Of course, the strength of edge effects diminishes as one moves deeper inside forests, but in addition, many edge phenomena vary markedly even within the same habitat fragment or landscape. Factors that might promote edge-effect variability include the age of habitat edges [22]–[25], edge aspect [26], [27], the combined effects of multiple nearby edges [28]–[31], fragment size [10], the structure of the adjoining matrix vegetation [32]–[34], seasonality [35], influxes of animals or plant propagules from surrounding degraded lands [9], [36]–[38], extreme weather events [39], [40], and fires [41], [42].

**Figure 1:**
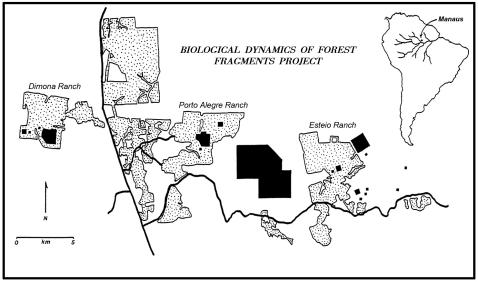
Study area in central Amazonia. Shaded blocks indicate locations of forest fragments and intact-forest controls used in the study. Stippled areas are cattle pastures or regrowth forest, while unstippled areas are intact forest. Thick, solid lines are roads.

In the Brazilian Amazon, up to 50,000 km of new forest edge is being created annually [43] as a result of rapid clearing and fragmentation of forests for cattle ranching, soy production, slash-and-burn farming, industrial logging, and wildfires [44]–[47]. For nearly three decades, we and our colleagues have studied edge effects in Amazonian forests as part of the world's largest and longest-running experimental study of habitat fragmentation [e.g. 5], [ 13]–[15], [ 17]–[19], [ 23], [ 24], [ 27]–[30], [ 32], [ 36], [ 38]–[41], [ 48]–[61](Figure 1). Here we evaluate factors that instigate variability in edge phenomena, focusing on ten edge-related changes in forest dynamics, plant community composition, invasive species, and carbon storage. Our findings prompt us to present a new hypothesis about the behavior of fragmented ecosystems that, if valid, will have key implications for biodiversity conservation.

## Results

### Variability in edge effects

Most of the edge-effect variables we evaluated (see Methods) exhibited pronounced spatial and temporal variability. For example, two of the most ecologically important parameters, the overall rates of tree mortality and recruitment, were spatially much more variable near forest edges (plot center<100 m from edge) than in forest interiors (>100 m from edge). Using standard deviations (SDs), among-plot variability was dramatically elevated for both mean mortality (*F_32,32_* = 3.29, *P* = 0.0006) and recruitment (*F_32,32_* = 8.13, *P*<0.0001; *F*-tests). These differences did not simply result from higher mean mortality and recruitment rates near edges (Figure S1), because coefficients of variation (CV), which adjust for differences in mean values, were also elevated near edges (mortality: 40.5% vs. 34.1%; recruitment: 47.9% vs. 27.9%).

**Figure 2:**
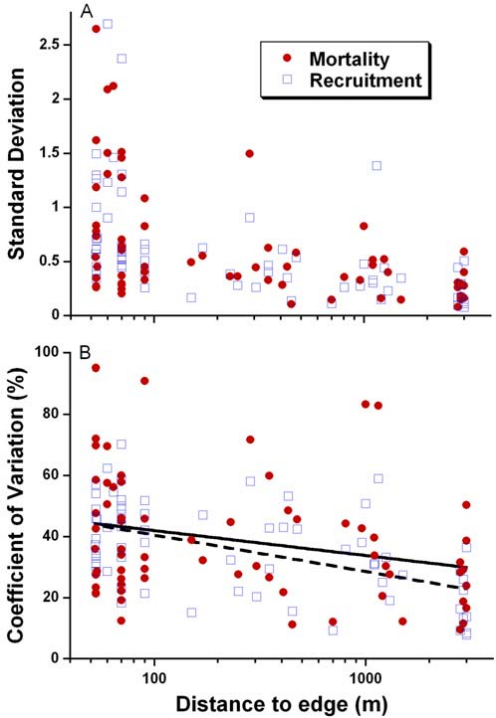
Pronounced temporal variability of tree mortality and recruitment rates near Amazonian forest edges. This variability is measured by (A) standard deviations and (B) coefficients of variation of among-census variation for 1-ha plots. In (B), the solid regression line is for mortality (*F_1,64_* = 5.68, *R^2^* = 8.2%, *P* = 0.02) and the dashed line for recruitment (*F_1,64_* = 27.30, *R^2^* = 29.9%, *P*<0.0001).

Tree mortality and recruitment rates were also temporally far more variable near edges. When among-census variation was quantified for each plot using SDs (Figure 2A), values for both mortality (*r_s_* = −0.440, *P* = 0.0002) and recruitment (*r_s_* = −0.670, *P*<0.00001) were sharply elevated near edges (Spearman rank correlations). CVs for mortality and recruitment also rose markedly nearer fragment margins (Figure 2B), indicating that both were temporally hyper-variable near edges.

Most other edge parameters exhibited similar trends. On average, tree densities fluctuated more dramatically over time near forest edges than in interiors (*P* = 0.0023, Mann-Whitney *U*-test), and these fluctuations were more spatially variable near edges (*F_32,32_* = 8.14, *P*<0.00001; *F*-test based on variances in CV values). Likewise, edge plots had sharply elevated spatial variability in overall tree-community change (*F_18,20_* = 17.76, *P*<0.0001), floristic trajectories 1 and 2 (both *F_18,20_*>9.08, *P*<0.0001), the abundance of pioneer and invasive trees (*F_32,32_* = 11.59, *P*<0.00001), and tree-species turnover (*F_18,20_* = 15.25, *P*<0.0001), relative to forest interiors (all *F*-tests comparing variances in edge vs. interior plots). Only liana abundance (*F_32,32_* = 1.35, *P* = 0.20) and the average rate of biomass change (*F_32,32_* = 1.19, *P* = 0.31) did not increase in spatial variability on edges relative to interiors, and even these had somewhat (17–19%) higher SDs on edges.

### Predictors of variability

Three variables, cattle ranch, distance to forest edge, and the number of nearby edges, were the most important predictors of spatial variation in edge phenomena, having significant effects on six, five, and two edge-effect variables, respectively (Table 1). Analyses explained 38–60% of the total variation in edge variables. As expected, edge phenomena increased in intensity closer to forest edges and with more nearby edges. In pairwise comparisons among the three cattle ranches, tree mortality and recruitment, fluctuations in tree abundance, and the abundance of pioneer and invasive trees were all significantly (*P*<0.05) higher in fragments at Dimona than Esteio, with Porto Alegre being intermediate (Porto Alegre also had significantly higher recruitment than Esteio; Tukey's tests). Soil factors, slope, and fragment area per se had no significant influence on edge-effect variables.

**Table 1:** Predictors of spatial variability in edge-effect parameters in fragmented and intact Amazonian forests, using general linear models.

Response Variable	Distance to edge	Number of edges	Area	Ranch	Soil sand content	Soil C content	Slope	Multiple R^2^ (%)
Tree mortality	**0.048**	0.468	0.098	**0.047**	0.261	0.076	0.086	50.8
Tree recruitment	**0.014**	0.499	**0.039**	**<0.001**	0.768	0.141	0.117	53.9
Biomass change	**0.010**	0.114	0.256	0.112	0.647	0.255	0.078	42.7
Variation in stem no.	**0.030**	0.834	0.114	**0.027**	0.783	0.218	0.179	38.1
Pioneer abundance	0.898	**0.012**	0.244	**0.001**	0.953	0.215	0.986	52.5
Liana abundance	**0.006**	**0.001**	0.400	0.108	0.468	0.075	0.988	39.6
Net floristic change	0.564	0.597	0.715	0.250	0.437	0.282	0.759	59.6
Floristic vector 1	0.583	0.549	**0.021**	0.207	0.838	0.752	0.861	49.0
Floristic vector 2	0.613	0.818	0.522	**0.002**	0.115	0.628	0.198	56.6
Species turnover	0.226	0.504	0.634	**0.049**	0.299	0.266	0.727	60.1

*Notes:* The *P* value for each predictor is for a full model that includes all predictors (significant *P* values are shown in bold). Predictors for each plot include distance to the nearest forest edge, the number of nearby forest edges, fragment (or reserve) area, the cattle ranch in which fragments were located, percent sand content, soil carbon content, and the mean slope. Analyses are based on 40 1-ha plots randomly stratified across the study area (overall floristic change, floristic vectors 1–2, species turnover) or on all 66 1-ha plots in the study (all other response variables).

**Figure 3:**
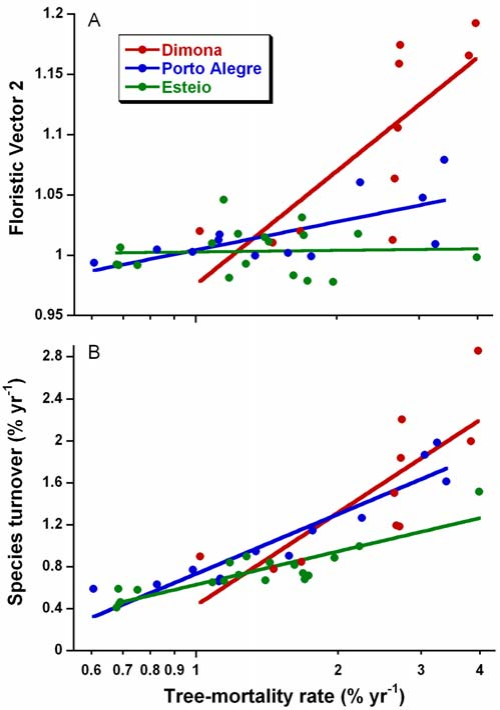
Ecological differences among forest fragments in three Amazonian cattle ranches. Differences among the ranches are shown for (A) floristic change (floristic vector 2) and (B) tree-species turnover, using the mean tree-mortality rate in each plot as a covariate.

Spatial and temporal variability in tree mortality evidently helps to drive variability in several other edge-effect phenomena. First, in simple (Bonferroni-corrected) correlations among the edge and predictor variables, tree mortality was strongly (*r*>0.60, *P*<0.00001) correlated with all other edge-effect parameters except liana abundance (Table S1). Second, when the GLM analyses were repeated but with tree mortality included as a potential predictor, model performance improved (explaining 42–91% of the variation in edge parameters) and tree mortality was a significant predictor for all edge variables except liana abundance (Table S2). Model improvement was greatest for four floristic variables (overall change in tree-community composition, floristic vectors 1 and 2, and the rate of tree-species turnover). Finally, for several edge-effect variables, such as floristic vector 2 (Figure 3A) and tree-species turnover (Figure 3B), tree mortality was a highly significant covariate when the variables were contrasted among cattle ranches.

**Figure 4:**
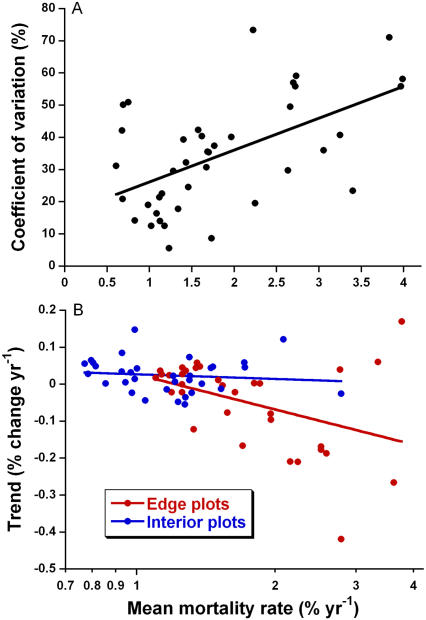
Temporal variability in tree mortality. (A) Tree mortality is highly episodic in Amazonian forest fragments, as shown by the strong relationship between the mean and CV of mortality rates, based on repeated censuses of 1-ha plots (*F_1,64_* = 13.17, *R^2^* = 17.1%, *P* = 0.0006). (B) Mortality rates decline with fragment age, but only among plots near forest edges (*F_1,31_* = 6.49, *R^2^* = 17.3%, *P* = 0.016), which have the highest overall mortality rates (there was no significant trend for forest-interior plots; *F_1,31_* = 0.42, *R^2^* = 1.3%, *P* = 0.52; linear regressions).

Given the apparently important impacts of tree mortality on forest ecology, we evaluated how tree-mortality rates vary over time, using data from the repeated censuses of our 66 plots. Two trends were apparent. First, although tree mortality was generally elevated near forest edges (Figure S1), it was also highly episodic, varying markedly among different census intervals. This is illustrated by the strong tendency for plots with high mean mortality rates (averaged over the entire study) to have significantly elevated CVs (Figure 4A). Second, mortality rates tended to decline somewhat with fragment age, at least among edge plots, which had the highest overall mortality rates (Figure 4B).

Collectively, these analyses suggest that elevated tree mortality partially drives changes in several other edge-effect phenomena, especially those relating to the intensity and pace of floristic change in fragments. Tree mortality is highly variable temporally and spatially, and tends to decline somewhat as fragments become older, especially among plots near forest edges. Although many edge phenomena were significantly affected by the proximity and number of nearby forest edges, as expected, they also differed to a surprisingly extent among the three large cattle ranches in our study area.

## Discussion

### Causes of variability in edge effects

For nearly three decades, we and our colleagues have studied ecological changes in forest fragments within a 1000-km^2^ experimental landscape, comprised by three large, isolated cattle ranches that were carved out of intact forest (Figure 1). Within these fragments, edge effects are clearly the dominant drivers of ecological change [5], [55], but the diverse edge phenomena we evaluated were often strikingly variable in space and time. Why?

Part of the pronounced spatial variability we observed arises from local factors such as the proximity and number of nearby forest edges (Tables 1, S1, and S2). Plots with two or more neighboring edges, such as those in small (1-ha) fragments and on the corners of larger fragments, have significantly greater tree mortality and biomass loss, fewer old-growth-tree seedlings [29], and higher abundances of pioneer and invasive tree species [30] and lianas [53], than do those with just one nearby edge. These patterns clearly support additive models of edge effects [28], which suggest that the intensity of edge phenomena is compounded by multiple nearby edges.

Edge age also influences edge effects. Edge-related tree mortality is especially intense in the first few years after edge creation (Figure 4)[5], [55], [62], in part because microclimatic changes are especially strong near newly formed edges, which are structurally open and thus highly permeable to the penetration of heat, light, and wind from outside degraded lands [24], [63]. In addition, most trees along newly formed edges are not physiologically acclimated to the sudden heat and desiccation stress, and many simply drop their leaves and die standing [5], [62]. Over time, the edge is partially sealed by proliferating vines and second growth, and microclimatic gradients lessen in intensity [7], [63]. Rates of tree death from physiological stress likely decline over time, both because older edges are less permeable and because trees that are poorly adapted for edge conditions (or in poor health generally) tend to die and be replaced by more desiccation-tolerant species [15]. These changes probably explain the moderate decline in tree-mortality rates with edge age observed in this study (Figure 4B).

Another driver of both spatial and temporal variability in edge effects is extreme weather events. The abrupt, artificial boundaries of forest fragments are especially vulnerable to windstorms, which can exert strong lateral-shear forces on exposed trees and create downwind turbulence for at least 2–10 times the height of the forest edge [64], [65]. In the Amazon, the most intense wind blasts come from convectional thunderstorms, which can cause severe but localized forest disturbance [66], [67]. Such windstorms are largely random events [66] that interact with local topography, leading to spatially complex patterns of forest disturbance [68]. Since our study commenced in 1979, fragments in the Dimona and, to a lesser extent, Porto Alegre ranches have been heavily damaged by windstorms, whereas those in Esteio ranch have remained largely unscathed [30], [38](Figure 1). These episodic wind disturbances cause considerable spatial and temporal variability in tree mortality and other correlated edge effects, such as floristic change and forest-biomass loss (Tables S1 and S2).

Periodic droughts also contribute to the temporal variability of edge effects, given the inherent vulnerability of rainforest edges to desiccation [23], [27]. Large areas of the Amazon are affected by the El Niño-Southern Oscillation (ENSO), which typically causes droughts or rainfall deficits at 3–7 year intervals. During the strong 1997 ENSO drought, dry-season rainfall was less than a third of average in our study area, and tree mortality and leaf-shedding by drought-stressed trees rose markedly near forest edges [40], [54]. In addition, destructive, edge-related forest fires proliferated dramatically across the Amazon [16], [42].

Finally, the structure and composition of the adjoining matrix vegetation can have a strong influence on edge effects. In our study area, forest edges adjoined by young regrowth forest, which helps to provide a physical buffer from wind and light, suffered less-intensive edge-related changes in microclimate [24] and lower tree mortality [32] than did those adjoined by cattle pastures. The species composition of the matrix vegetation is also important, because it influences the seed rain entering fragments [4], [27]. In our study area, tree species regenerating in fragments adjoined by *Vismia*-dominated regrowth were very different (more diverse and less dominated by the pioneer *Cecropia sciadophylla*) from those in fragments bordered by *Cecropia*-dominated regrowth [38]. Such differences can propel surprisingly rapid changes in the floristic composition of fragments [4], [15].

### The ‘Landscape-Divergence Hypothesis’

In this study, several of the factors described above manifested themselves as important differences in edge effects among our three large cattle ranches (Table 1, Figure 3). Such differences initially surprised us. Our three sprawling ranches (Figure 1) were carved out of the surrounding old-growth forest almost simultaneously, as part of the same government-sponsored program to promote large-scale cattle ranching in the central Amazon. The three ranches had broadly similar vegetation and climate (despite certain differences in soils, slope, and their initial tree-community composition; see Methods and Protocol S1). Moreover, given that our study is a carefully controlled experiment, none of the ranches was subject to various complicating pressures, such as wildfires, selective logging, and overhunting, that plague many human-dominated landscapes [2], [69]. Yet despite such similarities, the fragments within the three landscapes have undertaken remarkably different trajectories of change. Why have these landscapes diverged?

The reason is that even small initial differences among the ranches quickly multiplied into much larger differences. Parts of the Porto Alegre and Esteio ranches were cleared in 1983, when an early wet season prevented burning of the felled forest [48]. Tall and floristically diverse *Cecropia*-dominated regrowth quickly developed in these areas, whereas areas cleared in other years became cattle pastures or, eventually, scrubby *Vismia*-dominated regrowth [70]. The differing matrix vegetation had major impacts on both the dynamics and trajectories of floristic change [15], [30], [38] and the composition of faunal communities [36], [48] in nearby fragments. These differences were magnified by subsequent windstorms, which severely damaged some fragments at Dimona and to a lesser extent at Porto Alegre, yet left the Esteio fragments unscathed. Even identically sized fragments in the three ranches have had remarkably different dynamics (Figure S1) and trajectories of compositional change.

**Figure 5:**
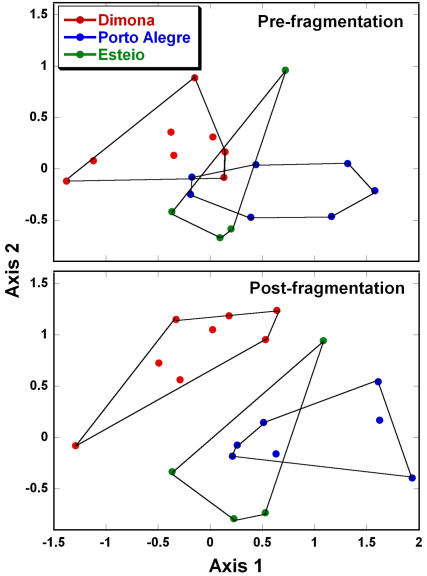
Increasing divergence of tree-community composition in three fragmented landscapes. Tree communities in forest-edge plots (<100 m from the nearest edge) are shown before forest fragmentation and 13–18 years after fragmentation, based on a single ordination of all plots and censuses in the study area. The ordination used importance values for all 267 tree genera found in the plots.

The apparently acute sensitivity of fragments to local landscape and weather dynamics—even within a study area as initially homogeneous as ours—prompts us to propose a new hypothesis about the functioning of fragmented ecosystems. We suggest that fragments within the same landscape will tend to have similar dynamics and trajectories of change in species composition, which will often differ from those in other landscapes. Over time, we believe, this process will act as a homogenizing force for fragments within the same landscape, and will promote increasing ecological divergence among fragments in different landscapes (as a corollary, fragments that experience similar matrix, disturbance, and environmental conditions are predicted to converge in composition, even if they are not in the same vicinity). This concept is illustrated by the rapidly changing tree communities in our study area, which appear to be diverging in composition among the three cattle ranches (Figure 5), and by other key differences in ecological dynamics among the ranches (Table 1, Figure 3).

This ‘landscape-divergence hypothesis’ can be contrasted with the principle of nested subsets [71], [72], which predicts that habitat fragments across a region will converge in species composition—regardless of their disturbance history or local landscape features. According to the nested-subsets concept, the biota in low-diversity fragments will comprise a proper subset of those in higher-diversity fragments or intact habitat. Although the predictions of the landscape-divergence and nested-subsets hypotheses differ markedly, they are not mutually exclusive: habitat fragments in different landscapes could increasingly diverge over time, but still support subsets of the same high-diversity species pool found in intact habitat. Landscape divergence might help to explain, for example, the weakly nested structure observed in some fragmented communities [see 72 and references therein].

If our hypothesis is correct, then different fragmented landscapes may tend to diverge not only in species composition but also in ecosystem functioning. Differences in characteristics such as forest dynamics, carbon storage, functional-guild composition, and species invasions could gradually accumulate over time, leaving an increasingly pervasive signature of divergence on community composition and functioning. In practice, however, discriminating the effects of landscape divergence from preexisting patterns of beta diversity may not be straightforward, at least in the absence of pre-fragmentation data. Statistical techniques such as additive partitioning [73], [74] might be useful for apportioning variation in species diversity within and among landscapes, and thus for contrasting certain predictions of the nested-subsets versus landscape-divergence hypotheses.

### Potential Implications

We conclude by highlighting three potential implications of our findings. First, the striking variability in edge effects we observed suggests that short-term or small-scale studies may fail to detect important edge phenomena, or may characterize them inadequately [7], [9]. In this study, our confidence was bolstered by the fact that we had pre-fragmentation data on tree-species distributions, stand structure, and biomass across our entire network of study plots. Even so, further replication would have been helpful for characterizing spatial variability in edge phenomena. Because of such inherent variability, it has been suggested that the known penetration-distance of edge effects should be doubled for management purposes [75], such as when designing buffer zones for nature reserves.

Second, our landscape-divergence hypothesis suggests that, rather than simply homogenizing biotas via selective extinctions, habitat fragmentation could also promote important landscape-scale differences among biotas. If so, this phenomenon should be incorporated into conservation planning, as it could imply, for example, that protected areas in different landscapes could preserve biologically and functionally different components of ecosystems.

Finally, our findings highlight the key impact of matrix vegetation on fragment dynamics [see also 76]. In the Amazon, among the worst (and unfortunately most common) land-use practices is one in which forest fragments are encircled by pastures, which are regularly burned by ranchers to control weeds and promote a flush of green grass for cattle. These fires destroy secondary vegetation and continually raze and re-open fragment edges, thereby maximizing the intensity of edge-related microclimatic stresses. Like a scab that is continually picked at, the forest edge cannot heal itself. During drought years, moreover, the rancher-lit fires can penetrate deep into fragments, greatly increasing forest degradation [41], [42]. In such contexts, protecting forest edges and their adjoining matrix is probably the single most important strategy for reducing the deleterious impacts of habitat fragmentation.

## Materials and Methods

### Study area and plots

The Biological Dynamics of Forest Fragments Project (BDFFP) is a 1000-km^2^ experimental landscape, dominated by non-flooded rainforest, located 80 km N of Manaus, Brazil (2°30′S, 60°W) at 50–100 m elevation (see Protocol S1 for details). The study area has three large (3,000–4,000 ha) cattle ranches, named Dimona, Porto Alegre, and Esteio, that contain a series of replicated forest fragments ranging from 1–100 ha in area. The fragments were created in the early-mid 1980s by felling and burning the surrounding forest to create cattle pastures. Nearby study sites in intact forest serve as experimental controls.

The three ranches in the study area are separated by expanses of primary forest and differ in certain respects [15], [30], [38], [48]. The ranches vary somewhat in slope, sand content, and soil-carbon content, and even before the fragments were created there were certain differences in tree abundance and species composition, but not tree-species richness, among the ranches (see Protocol S1). These initial disparities were greatly magnified by subsequent differences in land-use history among the ranches, which strongly influenced the amount and species composition of regrowth forest surrounding the fragments, and by wind disturbances, which had widely varying effects on the three ranches.

Prior to fragment isolation, standardized surveys of trees, mammals, birds, amphibians, many invertebrate groups, and other taxa were conducted in all fragment and control sites, and these and other taxa have since been monitored regularly. The ten edge-effect variables we evaluated were collected within a network of 66 1-ha permanent plots, arrayed across nine fragments and eight intact-forest sites. Plots were sampled at regular (typically 4–6 year) intervals, from the early-mid 1980s through 2004 (see Protocol S1). Nearly 1300 tree species or morphospecies have been identified in these plots.

### Edge-effect variables

For each plot, data were collected on (1) annual rate of tree mortality, (2) annual rate of tree recruitment, (3) the CV in tree density across censuses, (4) annual rate of change in aboveground tree biomass, (5) liana abundance, (6) overall density of pioneer and invasive tree species (belonging to the genera *Annona, Bellucia, Cecropia, Croton, Goupia, Jacaranda, Miconia, Pourouma,* and *Vismia*), (7) mean rate of tree-species turnover, (8) overall rate of change in tree-community composition (using Euclidean distances to measure change in importance values for 267 tree genera), and (9 and 10) two vectors of floristic change in the plots (using an ordination analysis to assess trajectories of floristic change; see Protocol S1). These ten variables describe edge-related changes in forest dynamics and carbon storage (parameters 1–4), the abundance of key plant-functional groups (parameters 5–6), and plant species composition (parameters 7–10) in each plot.

Previous studies [11]–[13], [47]–[51] have revealed that all of these parameters are significantly altered near forest edges, but did not explicitly evaluate patterns of spatial and temporal variability in the parameters. All ten edge parameters were used to assess spatial (among-plot) variability in edge phenomena. Temporal (within-plot) variability was also evaluated for two of the most important edge phenomena, tree mortality and recruitment rates, because values could be generated for the individual plot censuses. In addition to these new analyses of edge-effect variability, this study includes five years of previously unpublished forest-dynamics data for our plots.

### Predictor variables

We assessed the efficacy of seven key landscape, soil, and topographic factors to explain spatial variability in our edge-effect variables (see Protocol S1). These included (1) fragment/reserve area, (2) linear distance of each plot to the nearest forest edge, (3) the number of nearby forest edges, (4) cattle ranch (Dimona, Porto Alegre, Esteio), (5) soil percent sand content, (6) soil organic-carbon content, and (7) mean slope of each plot. Predictors 1, 3, and 4 were treated as categorical variables; all others were continuous.

### Data analysis

General linear models (GLM) were employed to assess effects of predictor variables on edge-effect parameters, using Systat version 10. None of the predictors was strongly (R^2^>50%) intercorrelated. Log and arcsine-squareroot transformations were used as needed to improve data normality. GLM performance was assessed by comparing standardized residuals to the fitted values and to each significant predictor.

## Supporting Information

Protocol S1 Description of Study Area and Methods Used to Quantify Edge-Effect and Predictor Variables
(0.04 MB DOC)

Table S1 Pearson Correlations Between Edge-Effect Parameters and Habitat Predictors
(0.04 MB DOC)

Table S2 Predictors of Spatial Variability in Edge-Effect Parameters, With Tree Mortality Included as a Potential Predictor
(0.04 MB DOC)

Figure S1 Long-term Average Rates of Tree Mortality and Recruitment, as a Function of Distance from Forest Edge
(0.03 MB DOC)
