# Supplementary material for: Habitat Fragmentation, Variable Edge Effects, and the Landscape-Divergence Hypothesis
Source: PLoS One. 2007 Oct 10;2(10):e1017. doi: 10.1371/journal.pone.0001017 (PMC1995757; doi:10.1371/journal.pone.0001017)
Supplement: Protocol S1 — Description of Study Area and Methods Used to Quantify Edge-Effect and Predictor Variables (0.04 MB DOC) [file pone.0001017.s001.doc]

**Protocol S1: Details on Study Area and Methods**

**Study area**

The topography of the BDFFP study area consists of flat or undulating plateaus interspersed by many steeply eroded gullies. Rainforests in the area are not seasonally inundated. Rainfall ranges from 1,900-3,500 mm annually with a pronounced dry season from June to October. The forest canopy is 30-37 m tall, with emergents to 55 m. Species richness of trees is very high and can exceed 280 species (≥ 10 cm diameter at breast height [dbh]) per hectare.

The study area is surrounded by large expanses (>200 km) of continuous forest to the west, north, and east. In the early-mid 1980s, a series of forest fragments ranging from 1-100 ha in area were isolated (by distances of 70-1,000 m) from the surrounding forest in the Dimona, Porto Alegre, and Esteio ranches by clearing the intervening vegetation to establish pastures. Fragments were fenced to prevent encroachment by cattle. Reserves ranging from 1-1,000 ha in area were delineated in nearby continuous forest to serve as experimental controls. Parts of the three ranches were soon abandoned, with *Cecropia*-dominated regrowth proliferating in areas that were cleared but not burned, and *Vismia*-dominated regrowth regenerating on pastures that were repeatedly burned [1].

The dominant soils in the study area are xanthic ferralsols. Ferralsols are widespread in the Amazon Basin, heavily weathered, and usually have a low base saturation. They are generally well aggregated, porous, and friable, with variable clay contents. Clay particles in ferralsols can form very durable aggregations, giving the soil poor water‑holding characteristics, even with high clay contents. Xanthic ferralsols in the Manaus area are derived from Tertiary deposits and are typically acidic and very poor in nutrients such as P, Ca, and K [2].

**Network of permanent plots**

Since 1980, a long-term study of tree-community dynamics and composition has been conducted in fragmented and continuous forests in the study area. Over 60,000 trees (≥ 10 cm dbh) are being monitored at regular (typically 4-6-year) intervals within 66 permanent, square 1-ha plots spanning the study area. Thirty-nine of the plots are located within fragments: four 1-ha fragments (4 plots), three 10-ha fragments (17 plots), and two 100-ha fragments (18 plots). The remaining 27 control plots are arrayed in nine reserves that roughly mimic the spatial arrangement of fragment plots, but in continuous forest. Plots within fragments and continuous forest are stratified so that both edge and interior areas are sampled.

All plots were initially censused between January 1980 and January 1987, then recensused 3-6 times, with the most recent recensus completed in mid-2004 (mean=5.4 censuses plot^-1^). During the initial census, trees in each plot were marked with numbered aluminum tags and mapped. During recensuses, all new trees (≥ 10 cm dbh) were mapped and marked, and all dead or severely damaged trees (with broken crowns or snapped boles) were recorded. On average, 95.3% of all trees in each plot have been identified to species or morphospecies level, using sterile or fertile material collected from each tree. Voucher specimens for each tree are maintained in the BDFFP Plant Collection, Manaus, Brazil.

**Edge-effect variables**

The ten edge-effect variables described changes in forest dynamics and carbon storage, the abundance of certain plant-functional groups, and plant species composition. Two key variables, the rates of tree mortality and tree recruitment, were used for evaluating both spatial (among-plot) and temporal (within-plot) variability in edge phenomena, because values were recorded both for individual plot censuses and averaged over the entire study for each plot. The remaining eight variables quantified mean rates of change or instability in each plot, averaged over the entire study, and thus could be used only for evaluating spatial (among-plot) variability in edge effects.

The edge-effect variables were generated using data collected from the early 1980s through either 2004 (mortality, recruitment, fluctuations in tree abundance) or 1999 (all other variables). Data on forest dynamics, biomass, and disturbance-adapted plants were collected in all 66 plots, whereas indices of floristic change were derived for a subset of 40 plots that were randomly stratified across all fragments and intact-forest reserves in the study area. For floristic analyses, a relatively small number of non-identified stems in each plot (4.7% on average) were excluded.

Four of the edge variables described forest and biomass dynamics. Annualized rates of tree mortality and tree recruitment were determined for each plot and census, using standardized logarithmic models. The mean rate of change in aboveground dry biomass (tons ha^-1^ yr^-1^) was estimated for each plot by calculating total tree biomass for each census (using dbh data and the allometric formula recommended by [3]), regressing the biomass values against time (number of years since the initial census), and then using the slope term as an estimate of biomass change. Fluctuations in tree abundance in each plot were quantified by calculating coefficients of variation (CV) in stem density across all censuses.

The next two variables described the abundance of plant-functional groups dominated by light-demanding, disturbance-adapted species. The total abundance of lianas was determined by counting all liana stems (≥ 2 cm dbh) in the final census of each plot. The abundance of pioneer and invasive trees was the percentage of all stems within nine early successional genera (*Annona, Bellucia, Cecropia, Croton, Goupia, Jacaranda, Miconia, Pourouma, Vismia*) in the final census of each plot. Of the 52 pioneer species found in these genera, at least a tenth rarely if ever occur in intact rainforest [4].

The remaining variables quantified changes in tree-community composition. The rate of tree-species turnover was calculated for each plot and census interval as ((Ne+Ng)/(No+Ng)) x 100, using data on 1162 tree species, where Ne=number of local extinctions during the interval, Ng=number of new species encountered during the interval, and No=number of species at the beginning of interval; values for each interval were then weighted by census duration and averaged [5]. The overall rate of change in tree-community composition was estimated by comparing the importance values of 267 tree genera between the initial and final censuses of each plot, using Euclidean distances [5].

Finally, an ordination analysis was used to identify edge-related trajectories of floristic change in the plots, based on importance values for all 267 tree genera [5]. In this analysis, plot samples were arrayed in positions in ordination space that reflected their relative floristic composition (using nonmetric multidimensional scaling [NMDS] with Sorensen’s distance metric), such that changes in the position of individual plots over time describe trajectories of change in floristic composition. Randomization tests were used to determine the number of significant axes in the analysis. For each plot and ordination axis, plot trajectories were calculated by regressing the ordination score for each plot census against time (number of years since the initial pre-fragmentation census) to determine the mean annual distance that the plot moved along each axis. The ordination analysis revealed three major gradients in floristic composition (axes 1-3) and explained 67% of the total variation in the dataset. The axis-3 trajectory did not differ significantly between edge and interior plots and was not considered further, but the two most important trajectories of floristic change were clearly associated with forest edges [5].

###### **Predictor variables**

###### We evaluated the efficacy of seven landscape, soil, and topographic factors to explain spatial variability in our response variables. Three landscape variables, fragment/reserve area, the distance of each plot to the nearest forest edge (measured from the center of the plot), and the number of nearby forest edges (<100 m from the plot center), describe the vulnerability of forests to edge and area effects. A fourth variable distinguished the three cattle ranches, which differed in the amount and type of regrowth forest present and in the intensity of past windstorms.

Two soil variables, organic-carbon content and sand fraction, describe potentially important attributes of forest soils, whereas slope is a key topographic variable. In our study area, flat areas tend to have high clay and organic-carbon contents, which are associated with relatively high (although still very modest) concentrations of important nutrients such as nitrogen and exchangeable bases. Lower slopes and valley bottoms typically have higher sand contents and lower nutrient concentrations [2]. Mean slope was determined by dividing each plot into 25 quadrats of 20 x 20 m each, measuring the maximum slope in each quadrat with a clinometer, and averaging these values. For the soil data, 9‑13 quadrats spanning the plot were selected for sampling; within each, 15 surface samples (top 20 cm below litter layer) were collected with a soil auger, and then bulked and subsampled. Textural analyses, using the pipette method, were used to determine percent sand content, whereas total organic carbon was determined by dry combustion. Data from the different quadrats were averaged for each plot.

**Differences among ranches**

Plots within the three cattle ranches exhibit some edaphic differences. Slope varies significantly among plots in the three ranches (*F_2,63_*=7.37, *P*=0.0013), with those in Dimona (*P*<0.01) and Esteio (*P*<0.05) being steeper than those in Porto Allegre. Soil-sand content also varies significantly (*F_2,63_*=3.22, *P*=0.047), being higher (*P*<0.05) in Dimona than Porto Allegre. Finally, soil-carbon context varies among the ranches (*F_2,63_*=6.34, *P*=0.0031), being higher in Porto Allegre than in Esteio (*P*<0.01)(all one-way ANOVAs with Tukey’s tests).

The three ranches also exhibited certain initial differences in tree communities, before the fragments were created. The overall density of tree stems varied significantly among the ranches (*F_2,63_*=16.66, *P*<0.0001), being higher in Dimona than in the other two ranches (*P*<0.001). Tree-community composition also varied significantly (*F_2,37_*=14.78, *P*<0.0001), with the Porto Allegre plots having significantly (*P*<0.001) higher scores on ordination-axis 1 (based on the NMDS analysis of all 267 tree genera, see above) than did the other ranches. However, neither ordination-axis 2 (*F_2,37_*=2.23, *P*=0.12) nor tree-species richness (*F_2,37_*=0.23, *P*=0.80) varied significantly among the three ranches. Tree-species richness also did not vary among the ranches when estimated using Fisher’s alpha-diversity index (*F_2,37_*=1.03, *P*=0.37), which is insensitive to variation in sample size (all one-way ANOVAS with Tukey’s tests).

**References**

1. Mesquita R, Ickes K, Ganade G, Williamson GB (2001) Alternative successional pathways in the Amazon basin. J Ecol 89: 528-537.
2. Chauvel A, Lucas Y, Boulet R (1987) On the genesis of the soil mantle of the region of Manaus, Central Amazonia, Brazil. Experientia 43: 234‑240.
3. Chambers JQ, Santos J, Ribeiro R, Higuchi N (2001) Tree damage, allometric relationships, and above-ground net primary production in central Amazon forest. For Ecol Manage 152: 73-84.

Laurance WF, Nascimento HEM, Laurance SG, Andrade A, Fearnside PM, et al. (2006) Rain forest fragmentation and the proliferation of successional trees. Ecology 87: 469-482.

1. Laurance WF, Nascimento HEM, Laurance SG, Andrade A, Ribeiro JELS, et al. (2006) Rapid decay of tree-community composition in Amazonian forest fragments. Proc Nat Acad Sci USA 103: 19010-19014.
