# Supplementary material for: Habitat Fragmentation, Variable Edge Effects, and the Landscape-Divergence Hypothesis
Source: PLoS One. 2007 Oct 10;2(10):e1017. doi: 10.1371/journal.pone.0001017 (PMC1995757; doi:10.1371/journal.pone.0001017)
Supplement: Table S1 — Pearson Correlations Between Edge-Effect Parameters and Habitat Predictors (0.04 MB DOC) [file pone.0001017.s002.doc]

**Table S1** Pearson correlations between edge-effect parameters and landscape, soil, and topographic predictors in fragmented and intact Amazonian forests.

_______________________________________________________________________

| Response  variable | Distance  To edge | No. of  edges | Area | Soil sand  content | Soil C  content | Slope | Tree  mortality |
| --- | --- | --- | --- | --- | --- | --- | --- |
| Tree mortality | **-0.522** | **0.536** | **-0.432** | 0.203 | -0.140 | 0.183 | ----- |
| Tree recruitment | **-0.470** | **0.522** | **-0.400** | 0.078 | -0.010 | 0.033 | **0.885** |
| Biomass change | **0.487** | **-0.431** | **0.514** | -0.087 | -0.030 | -0.089 | **-0.711** |
| Variation in stem no. | **-0.396** | **0.461** | -0.317 | 0.092 | -0.066 | 0.033 | **0.768** |
| Pioneer abundance | -0.356 | **0.556** | -0.360 | 0.071 | -0.055 | 0.118 | **0.729** |
| Liana abundance | -0.025 | 0.238 | -0.117 | -0.120 | -0.052 | -0.122 | 0.254 |
| Net floristic change | **-0.639** | **0.606** | **-0.620** | -0.011 | 0.072 | 0.178 | **0.877** |
| Floristic vector 1 | -0.428 | 0.403 | -0.423 | -0.123 | 0.169 | 0.036 | **0.666** |
| Floristic vector 2 | -0.429 | 0.392 | -0.427 | -0.016 | -0.025 | 0.206 | **0.605** |
| Species turnover | **-0.626** | **0.519** | **-0.528** | -0.006 | 0.086 | 0.056 | **0.900** |

_______________________________________________________________________

*Notes:* Signficant correlations are shown in bold, using a Bonferroni-adjusted alpha value (*P*=0.0025). Analyses are based on 40 1-ha plots randomly stratified across the study area (overall floristic change, floristic vectors 1-3, species turnover) or on all 66 1-ha plots in the study (all other response variables).
