# Supplementary material for: Habitat Fragmentation, Variable Edge Effects, and the Landscape-Divergence Hypothesis
Source: PLoS One. 2007 Oct 10;2(10):e1017. doi: 10.1371/journal.pone.0001017 (PMC1995757; doi:10.1371/journal.pone.0001017)
Supplement: Table S2 — Predictors of Spatial Variability in Edge-Effect Parameters, With Tree Mortality Included as a Potential Predictor (0.04 MB DOC) [file pone.0001017.s003.doc]

**Table S2** Predictors of spatial variability in edge-effect parameters in fragmented and intact Amazonian forests, with tree mortality included as a potential predictor in GLM analyses.

___________________________________________________________________________

| Response  variable | Distance  To edge | No. of  edges | Area | Ranch | Sand  content | Soil C  content | Slope | Tree  mortality | Multiple  R^2^ (%) |
| --- | --- | --- | --- | --- | --- | --- | --- | --- | --- |
| Tree recruitment | 0.135 | 0.896 | 0.079 | **<0.001** | 0.186 | 0.889 | 0.877 | **<0.001** | 88.4 |
| Biomass change | 0.096 | **0.008** | 0.18 | 0.733 | 0.720 | 0.973 | 0.406 | **<0.001** | 67.0 |
| Variation in stem no. | 0.252 | 0.866 | 0.572 | 0.312 | 0.559 | 0.926 | 0.771 | **<0.001** | 64.0 |
| Pioneer abundance | 0.197 | **0.006** | 0.379 | **0.031** | 0.445 | 0.845 | 0.215 | **<0.001** | 69.8 |
| Liana abundance | **0.003** | **<0.001** | 0.586 | 0.279 | 0.355 | 0.151 | 0.769 | 0.187 | 41.6 |
| Net floristic change | 0.687 | 0.914 | 0.908 | 0.449 | 0.406 | 0.882 | 0.299 | **<0.001** | 82.1 |
| Floristic vector 1 | 0.766 | 0.395 | 0.056 | 0.537 | 0.944 | 0.766 | 0.920 | **0.005** | 62.6 |
| Floristic vector 2 | 0.939 | 0.758 | 0.736 | **0.006** | 0.115 | 0.969 | 0.111 | **0.027** | 64.5 |
| Species turnover | 0.662 | 0.372 | 0.071 | **0.012** | 0.113 | 0.956 | 0.793 | **<0.001** | 90.7 |

___________________________________________________________________________

*Notes:* The *P* value for each predictor is for a full model that includes all predictors (significant *P* values are shown in bold). Analyses are based on 40 1-ha plots randomly stratified across the study area (overall floristic change, floristic vectors 1-2, species turnover) or on all 66 1-ha plots in the study (all other response variables).
