# Supplementary material for: Habitat Fragmentation, Variable Edge Effects, and the Landscape-Divergence Hypothesis
Source: PLoS One. 2007 Oct 10;2(10):e1017. doi: 10.1371/journal.pone.0001017 (PMC1995757; doi:10.1371/journal.pone.0001017)
Supplement: Figure S1 — Long-term Average Rates of Tree Mortality and Recruitment, as a Function of Distance from Forest Edge (0.03 MB DOC) [file pone.0001017.s004.doc]

**Figure S1** Long-term average rates of (A) tree mortality and (B) tree recruitment in 66 1-ha plots in fragmented and intact Amazonian forest, as a function of distance from forest edge.
